# Stability of Fly Maggot Peptides and Its Alleviating Effect on Lipopolysaccharide Combined with Hemocoagulase Oxidative Stress in Arbor Acres Chicks

**DOI:** 10.3390/vetsci11100470

**Published:** 2024-10-01

**Authors:** Qin Wang, Pingfei Qiu, Zeru Peng, Junlong Wu, Ruiying Bao, Liangmin Huang, Xiaochun Li, Huiyu Shi, Haiwen Zhang, Xuemei Wang

**Affiliations:** Animal Nutrition, Reproduction and Breeding Laboratory, Department of Animal Science, School of Tropical Agriculture and Forestry, Hainan University, Danzhou 571737, China; wangqin20210327@163.com (Q.W.); qpf773@163.com (P.Q.); 13134680495@163.com (Z.P.); wjl13976407172@163.com (J.W.); baoruiying524@163.com (R.B.); mlh9809@163.com (L.H.); l_x_chun@163.com (X.L.); shihuiyu2017@163.com (H.S.); hwzhang@hainanu.edu.cn (H.Z.)

**Keywords:** fly maggot peptide, stability, broilers chicken, oxidative stress, antioxidant, growth performance

## Abstract

**Simple Summary:**

Considering the issue of antibiotic residues, developing equivalent alternatives in the context of banning antibiotics is key. Peptides are considered a satisfactory option, and the current study aims to demonstrate the effect of prophylactic fly maggot peptides on oxidative stress. Oral administration of fly maggot polypeptide can improve the oxidative stress induced by lipopolysaccharide combined with hemocoagulase, and improve the growth performance, antioxidant function, immune function, and intestinal barrier function of broilers.

**Abstract:**

Recently, there has been fast-growing interest among researchers in discovering bioactive peptides from insects and evaluating their potential applications in livestock production. The present study aimed to assess the antioxidant properties and stability of fly maggot peptide (FMP) and its effects on Arbor Acres (AA) broilers’ oxidative stress induced by lipopolysaccharide (LPS) and hemocoagulase (HC). A total of 108 one-day-old AA broilers were randomly divided into six groups: CG (normal saline, basal diet), DG (LPS + HC, basal diet), VG (DG + vitamin C 50 ug/kg), LPG (DG + FMP 5 mg/kg), MPG (DG + FMP 15 mg/kg), and HPG (DG + FMP 25 mg/kg). The results showed that the addition of FMP to the diet promoted LPS+ HC-induced increases in average daily gain (ADG), total superoxide dismutase (T-SOD), total antioxidant capacity (T-AOC). Meanwhile, FMP regulated the intestinal morphology. Additionally, FMP decreased the increase in the contents of malondialdehyde (MDA), the relative weight of immune organs, interleukin-1β (IL-1β), interleukin-6 (IL-6), and tumor necrosis factor-α (TNF-α). In conclusion, this research suggested that the addition of FMP can relieve the LPS+ HC-induced oxidative stress of AA broilers and the recommended dose of FMP is 25 mg/kg. This study presents a theoretical foundation for the addition of an FMP supplement for the purpose of protecting broilers’ growth.

## 1. Introduction

Poultry meat and eggs are key foods to meet the dietary needs of the world’s growing population. Birds in large-scale intensive poultry production are subject to a variety of stressors and diseases, resulting in significant economic losses. There is evidence that most of the stresses found in the poultry sector (nutritional, intensive, physiological/pathological, or environmental factors) cause oxidative stress [1]. Oxidative stress begins with an imbalance between reactive oxygen species (ROS) production and antioxidant defenses [2]. Oxidative stress can lead to severe broiler injuries, such as decreased growth performance, disruption of antioxidant defenses, organic damage [3], and impaired host immune response [4]. Oxidative stress is linked to inflammation, which can cause intestinal mucosal damage and epithelial dysfunction [5,6]. Physiological ROS levels are required for the proper functioning of cells, tissues, and organisms [7]. Therefore, it is important to protect the body from oxidative stress and the body’s response to oxidative stress.

There is a current trend towards using natural substances with antioxidant properties that are traditionally used in medicine for treating or preventing various diseases. This shift is particularly noteworthy, as the use of antibiotics as growth promoters has been prohibited. Alternatives such as probiotics [8,9], prebiotics [10], herbal products [11], and bioactive peptides [12] are being introduced into poultry feed. Bioactive peptides refer to peptides that have biological functions in addition to their nutritional value [13]. Bioactive peptides, derived from food through enzymatic digestion and including substances like deer antler velvet, forest frog skin, pig skin, marine fish, etc., possess anti-inflammatory, antioxidant, and growth-promoting properties for animals. However, insect-derived bioactive peptides have better biological activity (antioxidant properties [14,15], antihypertensive activity that inhibits angiotensin-converting enzyme (ACE) production [16], antidiabetic activity and anti-inflammatory activity [17], and immunomodulatory effects [18]). Nevertheless, the impact of processing techniques and storage environments on peptides’ antioxidant properties remains largely unexplored. Amino acids in peptides consist of intricate components that may undergo degradation via deamidation reaction, oxidation, hydrolysis, and cyclization in the course of processing and storage, potentially diminishing their antioxidant properties [19]. Presently, it is essential to investigate the biological activity of antioxidant agents in living organisms. Given the significant role of the human gastrointestinal tract in oxidation, in vitro tests employing stimulated gastrointestinal digestion facilitate the preliminary evaluation of antioxidant peptides.

Fly maggots have long been used in medicine [20], and the adult stage is capable of biological disintegration due to its robust reproductive abilities and adaptability. More significantly, fly maggots, the larvae of *Musca domestica*, are protein-enriched and contain some bioactive components, such as polypeptides, and the hydrolysates can be used as a source of protein. According to a recent report, *Musca domestica* maggot polypeptide may reverse endothelial cell dysfunction in atherosclerotic lesions [21]. Furthermore, several scientists investigated active ingredients in maggots that exhibited exceptional antibacterial properties; these chemicals were identified as polypeptides and offered an alternative to antibiotic resistance [22]. Few studies report peptides acting as natural antioxidants that protect homeostasis from pathogenic factors [23,24]

Oxidative stress frequently results in decreased feed intake, slower development rates, and metabolic disorders, all of which have a significant negative financial impact on animal husbandry. Lipopolysaccharide (LPS) is one of the major components on the outer membrane of the cell wall of Gram-negative bacteria. LPS possesses active sites that are capable of activating the innate immune response that is the main inducement for acute infections and inflammatory responses [25,26]. Several studies showed that inflammation in many tissues can be triggered by LPS [27,28], including spleen [29] and liver [30]. However, a report found that the poultry showed considerable resistance to LPS and the administered dose failed to cause normal pathological responses [31]. In the present study, hemocoagulase (HC) and LPS were jointly administered via neck and enterocoelia, respectively, ensuring inflammatory responses in chicks, which had been carried out in a trial test before this study.

In previous studies, fly maggots have been shown to be an excellent protein feed for animals. Antibacterial, anti-inflammatory, and immunomodulatory effects have been reported in farm animals, but the antioxidant activity and stability of fly maggot peptide (FMP) and the effect of the addition of fly maggot antioxidant peptide to broiler diets on oxidative stress is unclear. Thus, the antioxidant effects and stability of fly maggot peptide and different concentrations of FMP for the alleviation of oxidative stress induced by HC and LPS on AA chickens were explored in this study.

## 2. Materials and Methods

### 2.1. Materials

Fly maggot larvae were purchased from Hainan Huanran Technology and Biological Co., Ltd., Haikou, Hainan. Arbor Acres (AA) broiler chicks came from Emerging Dahua Nong Poultry Egg Co., Ltd., Guangdong, China. The list of abbreviations in this manuscript is shown in Supplementary Table S1.

### 2.2. Bioactive Peptides

#### 2.2.1. Source

The FMP used in this study was obtained from our previous research. Briefly, the fly maggot larvae were homogenized, the pH was adjusted, and hydrolysis took place (pepsin and trypsin at 40 °C with pH 5.0 for 3 h). After inactivating the hydrolase, the processed homogenate was filtered and centrifuged at 5000 rpm for 15 min. The collected supernatant was concentrated using a rotary evaporator (Buchi Labortechnik AG, Shanghai, China) and lyophilized to obtain a polypeptide powder (with a purity of 69.6%).

#### 2.2.2. Determination of Antioxidant Activities of FMP

The antioxidant activity of FMP was determined using a detection kit. The total antioxidant capacity (DPPH, DPPH-2-D) was determined by combining FMP with 0.2 mmol/L DPPH ethanol working solution, which was then stored in the dark. The absorbance at 515 nm was measured using a UV-Vis spectrophotometer (Beijing Ruili Analytical Instrument Co., Ltd., Beijing, China, UV-1801).

According to the manufacturer’s instructions for the use of the detection kit, 5 μL of different concentrations of the mixture was added to each of the assay wells. The samples were mixed with 180 μL of FRAP (ferric ion-reducing antioxidant power) working solution and kept for 5 min at 37 °C. The absorbance of the reaction mixture was then recorded at 593 nm. The standard curve was prepared using FeSO_4_ ranging from 0.15 to 1.5 mM. The activity was expressed by FeSO_4_ values, which were calculated using standard curves.

#### 2.2.3. Stability of Hydrolysate of FMP

The DPPH· scavenging activity (%) of FMP at 10 mg/mL was measured at the set conditions to evaluate their stability. The thermostability of FMP at 25, 40, 60, 80, or 100 °C for 120 min was analyzed in a water bath. The influences of pH values of 2–10 were employed to estimate the acid and alkali stability properties of FMP at 25 °C, and the detecting time was set to 120 min. The influence of simulated GI digestion on the stability of FMP was evaluated by the two-stage digestion model. In short, FMP was separately treated with pepsin for 120 min and trypsin for 120 min.

(1) $$The\;antioxidant\;activity\;retention\;rate=\frac{A_{0}}{A_{1}}\times 100$$

$A_{0}$: The antioxidant activity of FMP.
$A_{1}$: Antioxidant activity of FMP after temperature or acid–base conditions or simulated GI digestion.

### 2.3. Animal Experiment

After four days of adaptation, 108 five-day-old AA broiler chicks with an initial body weight (BW) of 58.9 ± 3.1 g were randomly equally divided into six treatments of three replicates, each with six birds per replicate, which was approved by the Institutional Review Board of the Ethical Committee of the Hainan University (Haikou, China, permit number HNUAUCC-2022-00083). All the chicks were housed in the Laboratory Animal Center, Hainan University, with a 23 h light and 1 h dark cycle at 23 ± 2 °C. The chicks were provided with free access to water and feed.

The basal diet was commercial feed, and the nutrient requirements for the brooding period (days 1–21) were as recommended by the NRC (1994) and “Chicken Breeding Standard: NY/T33-2004”.

#### 2.3.1. Treatments and Sample Collection

The treatment groups are as follows: (1) control group (CG): oral administration of sterile saline for 3 days, followed by injection, respectively, once with sterile saline (0.2 mL) via intraperitoneal and subcutaneous (neck) routes on day 4; (2) damage group (DG): oral administration of sterile saline for 3 days, followed by injection, respectively, once with LPS (Beijing Solarbio Science & Technology Co., Ltd., Beijing, China) and HC (Jinzhou Aohong Pharmaceutical Co., Ltd., Jinzhou, China) via intraperitoneal and subcutaneous (neck) routes on day 4; (3) vitamin C group (VG): oral administration of 50 μg/kg BW of vitamin C solution for 3 days, followed by injection, respectively, once with LPS and HC via intraperitoneal and subcutaneous (neck) routes on day 4; (4) 5 mg/kg polypeptides group (LPG): oral administration of 5 mg/kg BW of polypeptide solution for 3 days, followed by injection, respectively, once with LPS and HC via intraperitoneal and subcutaneous (neck) routes on day 4; (5) 15 mg/kg polypeptides group (MPG): oral administration of 15 mg/kg BW of polypeptide solution for 3 days, followed by injection, respectively, once with LPS and HC via intraperitoneal and subcutaneous (neck) routes on day 4; and (6) 25 mg/kg polypeptides group (HPG): oral administration of 25 mg/kg BW of polypeptide solution for 3 days, followed by injection, respectively, once with LPS and HC via intraperitoneal and subcutaneous (neck) routes on day 4. Herein, intraperitoneal and subcutaneous administration of 0.2 mL of sterile saline in the absence of 5 μg/kg BW of HC and 150 U/kg BW of LPS, respectively [30]. Oral administration of 0.2 mL of sterile saline in the absence of 50 μg/kg BW of vitamin C (Shandong West Asia Chemical Co., Ltd., Linyi, China) or 5/15/25 mg/kg BW of polypeptides, respectively.

Blood samples were obtained by the heart punctures on day 1, day 3, and at the end of the experiment. Six chicks per treatment were chosen to collect blood samples (0.2 mL per chick) on day 1 and day 3, respectively (oxidative stress for 12 h and 72 h). The blood samples were centrifuged at 4500 rpm for 10 min at 4 °C to obtain supernatant and then stored at −80 °C for future analysis. All chicks were weighed and then sacrificed after carbon dioxide anesthesia on day 5. The liver, bursa of Fabricius, spleen, duodenum, jejunum, and ileum were sampled for future analysis.

#### 2.3.2. Growth Performance, the Relative Weight of the Immune Organ, and Rectal Temperature Detection

The BW of chicks was recorded on days 1, 3, and 5. Feed intake and leftovers for each cage were recorded daily. The spleen and bursa of Fabricius were weighed. The organ index (organ weight (mg)/broiler weight (g)) was calculated. Rectal temperature was detected after administering HC and LPS at 3, 6, 12, and 24 h using an electronic thermometer (Qingdao Yasee Medical Equipment Co., Ltd., Qingdao, China).

#### 2.3.3. Antioxidant and Inflammation Parameters

The total antioxidant capacity (T-AOC, A015-3-1), the activities of T-SOD (A001-1-2), GSH-Px (A005-1-2), and the content of MDA (A003-1-2) in the serum were measured with corresponding assay kits (Nanjing Jiancheng Bioengineering Institute, Nanjing, China).

The concentrations of IL-1β (ML-12), IL-6 (ML-13), TNF-α (ML-11), and IL-10 (ML-14) in livers were determined through an ELISA kit (Shanghai Enzyme-linked Biotechnology Co., Ltd., Shanghai, China). Protein normalization was used for comparison between samples. The BCA kit was used to determine the concentration (A045-4-2).

#### 2.3.4. Histological Analysis

For histological analysis, 5 chickens were randomly selected per replicate. The duodenum, jejunum, and ileum were sampled and fixed in 4% paraformaldehyde for 24 h. Then, the samples were sectioned and stained with hematoxylin and eosin (Beijing Solarbio Science & Technology Co., Ltd., Beijing, China) as described in a previous study [32]. The sections were observed under different magnifications using a Leica NEWDM 4500BR microscope (Leica, Frankfurt, Germany). ImageJ 1.54 g software was used to collect images; villus height (VH) and crypt depth (CD) were measured.

### 2.4. Statistical Analysis

All data are presented as the average of the pooled SEM values. Statistical analysis was performed using SPSS 26.0 for Windows (SPSS Inc., Chicago, IL, USA). The difference between treatment groups was assessed by the Shapiro–Wilk test of normal distribution followed by one-way ANOVA and Tukey’s test. A *p*-value less than 0.05 was considered statistically significant.

## 3. Results

### 3.1. Antioxidant Activities of FMP

**Figure 1:**
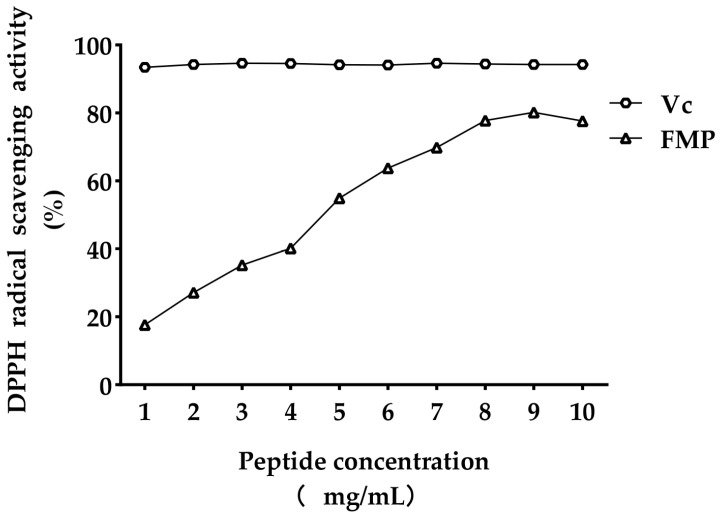
Scavenging ability on DPPH.

As shown in Figure 1, in the concentration range of 1–10 mg/mL, the scavenging capacity of DPPH radicals increased with the increase in concentration, and the scavenging capacity reached more than 80%. After statistical analysis using GraphPad Prism 9.0.0 (121) software, the half inhibitory concentration (half maximal inhibitory concentration, IC50) of DPPH radicals cleared by FMP was 4.186 mg/mL.

### 3.2. Stability of Antioxidant Peptides from FMP

**Figure 2:**
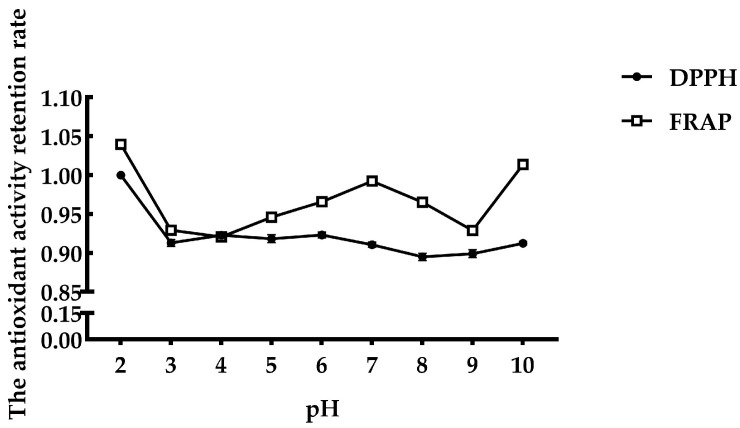
Effect of pH on FMP.

The antioxidant activity of FMP under different pH values is shown in Figure 2. Peptides at pH = 2 exhibited the strongest DPPH radical scavenging activity, and there was no significant decrease with increasing pH. The Fe^2+^-chelating ability showed a fluctuating trend but was not significantly affected by pH. The retention rate of antioxidant activity was always above 90%.

**Figure 3:**
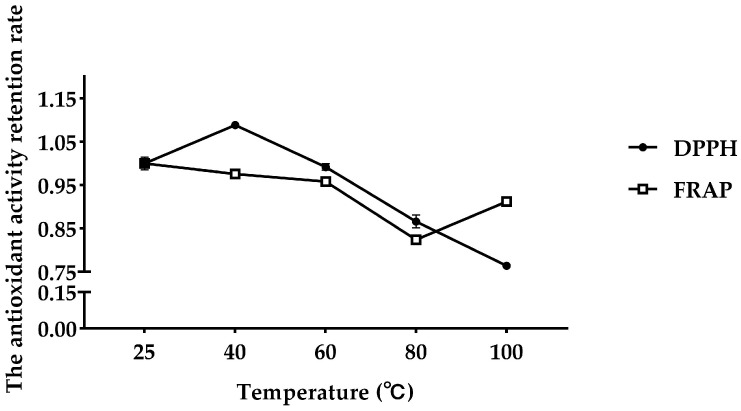
Effect of temperature on FMP.

It can be seen that in the range of 25~100 °C, the antioxidant peptide of fly maggots gradually decreases with the increase in temperature, and the antioxidant activity retention rate of DPPH gradually decreases. Treated at 80 °C for 2 h, the antioxidant activity retention rate of DPPH was 88–90% (Figure 3). The reducing force of FRAP decreased, and after 2 h of treatment at 100 °C, the reducing force increased, and the antioxidant activity retention rate was above 90% (Figure 3).

**Figure 4:**
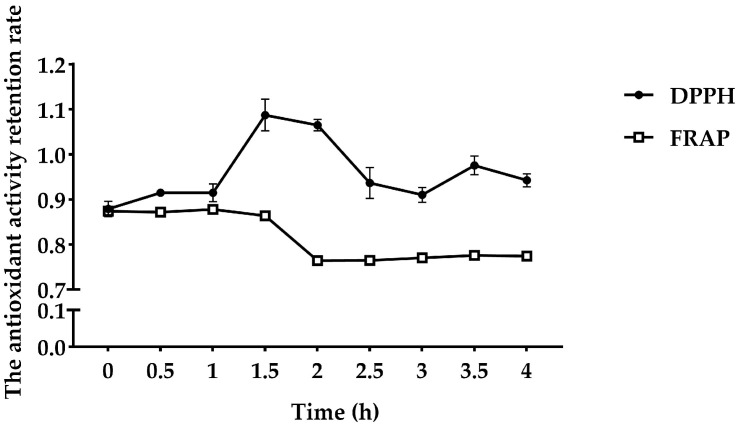
Effect of the simulated gastrointestinal tract enzyme line on FMP in vitro.

As shown in Figure 4, the retention rate of the maggot polypeptide to DPPH free radicals was 0.88 during simulated gastric digestion using pepsin (1–2 h). After pepsin digestion for 1.5 h, the retention rate of clearance capacity increased to 1.09. The reducing power before digestion was 0.87. After 2 h of simulated gastric digestion, the reducing power decreased to 0.76. During simulated intestinal digestion (2–4 h), FMP scavenging of DPPH free radicals decreased after trypsinization for 0.5 h, but high antioxidant activity was maintained. After trypsinization, the reducing power of FMP was maintained at 0.77.

### 3.3. Effect of Fly Maggot Polypeptide on Rectal Temperature, Growth Performance, and Relative Weight of Immune Organ

Rectal temperature was measured at 3, 6, 12, and 24 h after inducing OS, which suggested that the rectal temperatures in the CG were significantly lower than those of other groups at 6 h, as shown in Table 1 (*p* < 0.05). Similarly, compared with the CG, the rectal temperatures of the DG, VG, LPG, and HPG chicks were significantly increased at 12 h (*p* < 0.05). At 24 h, a significant anus temperature decrease can be seen in the MPG, compared to the CG (*p* < 0.05).

As shown in Table 2, the initial and final weight of chicks show no significant difference among groups (*p* > 0.05). However, a significant increase in average daily gain (ADG) was found in the HPG and VG compared with the DG (*p* < 0.05).

Table 3 illustrates the relative weight of the bursa of Fabricius and the spleen, and the results indicated that compared with the DG, the relative weight of the spleen significantly decreased in the CG, VG, and HPG (*p* < 0.05). Herein, the relative weight of the bursa of Fabricius showed no significant changes among groups (*p* > 0.05).

### 3.4. Effect of FMP on Serum Indexes at Different Time Periods

As shown in Table 4a, at 12 h, serum MDA levels were significantly reduced in the VG and MPG compared with the MG (*p* < 0.05). The DG showed no significant serum GSH-Px differences from the CG, LPG, MPG, and HPG (*p* > 0.05), but the serum GSH-Px was higher in the MPG than in the DG. The T-AOC values showed significant differences between the MG and the CG (*p* < 0.05). After the addition of FMP, the T-AOC enzyme activity in the serum returned to its normal state.

As shown in Table 4b, at 72 h, similarly, the highest MDA level was found in the DG with a significant difference compared to any group (*p* < 0.05). The CG and MPG had significantly lower GSH-Px compared to the DG (*p* < 0.05).

As shown in Table 4c, at 5 d, the highest level of MDA was still found in the DG and was markedly higher than that of the VG and MPG (*p* < 0.05). Additionally, higher T-SOD activity was observed in the MPG and HPG by contrast with the CG, VG, and LPG (*p* < 0.05).

### 3.5. Effect of FMP on Inflammatory Cytokines of the Liver

The results of inflammatory cytokines of the liver are shown in Table 5, which indicated that the CG, VG, LPG, MPG, and HPG had decreased IL-1β content in the liver with a significant change compared to the DG (*p* < 0.05). OS markedly increased IL-6 content in the DG, which suggests higher values than that of the CG (*p* < 0.05), and compared with the DG, the content was reversely decreased in the VG, LPG, MPG, and HPG (*p* < 0.05). The CG, LPG, MPG, and HPG had significantly decreased IL-10 content since the higher value was measured in the DG (*p* < 0.05). The TNF-α level in the DG was significantly higher than that of the VG, LPG, MPG, and HPG (*p* < 0.05).

### 3.6. Effect of FMP on Villus Height, Crypt Depth, and VCR of Small Intestine

There was an increase in the villus height of the duodenum (Table 6a) in the CG by contrast with the DG (*p* < 0.05). The depth of the crypt in the CG was significantly higher than that in the VG, LPG, and MPG (*p* < 0.05). There were no significant differences in VCR between groups (*p* > 0.05). There was an increase in jejunum (Table 6b) villus height in the LPG, MPG, and HPG compared with that of the DG (*p* < 0.05). As for the ileum (Table 6c), the HPG had markedly increased villus height by contrast with the DG (*p* < 0.05). However, the villus height of the HPG was higher than that of the LPG, MPG, and VG (*p* < 0.05). Compared to the DG, the VCR was notably significantly increased in the HPG (*p* < 0.05).

## 4. Discussion

The antioxidant activity of FMP increased with increasing concentrations. FMP is a good antioxidant compound with radical scavenging activity, although the activity is lower than that of vitamin C. When the concentration reached 1 mg/mL, the antioxidant activity decreased. The potential reasons for this decrease are as follows: Peptide composition and seriality of amino acids have a direct impact on antioxidant function. FMP may contain some amino acids with antioxidant activity (e.g., His, Tyr, Met, and Cys) [33,34,35,36]. A peptide’s structure and assay method determine its antioxidant characteristics [37]. FMP was found to have a somewhat mitigating effect on H_2_O_2_-induced oxidative stress damage in our earlier study. FMP may also lower intracellular ROS and MDA levels, increase the activity of endogenous antioxidant enzymes, and thus significantly protect against cellular oxidative stress damage, potentially serving as an antioxidant [38].

Thermal and acid/alkali stability are important processing parameters for peptides applied in functional products to lengthen the product shelf-life and expand their use in various fields. The temperature stability profiles are important, as these stability profiles will determine the optimal storage conditions for antioxidant peptides and whether they are indeed compatible with thermal treatments in food processing. Previous studies have reported on how temperature [39] and pH [40] could affect peptides’ antioxidant potential. In the study of the antioxidant activity of FMP, as the temperature increased from 25 to 60 °C, the DPPH radical scavenging activity increased, and the total FRAP antioxidant capacity remained largely unchanged. There was a decline in antioxidant activity of FMP between 60 and 100 °C. Moreover, these trends were in agreement with data from previous studies, demonstrating that most proteins were denatured at temperatures from 60 °C to 100 °C [41]. These trends may be due to the absence of tertiary and quaternary structures in short-chain and low-molecular-weight peptides. However, these peptides can still form secondary structures, which are the key factors affecting antioxidant activity. High treatment temperatures and extended exposure time may change the secondary structure of the antioxidant peptide, leading to a large decrease in activity. At pH 2, the peptide exhibited the highest DPPH scavenging activity, which decreased with rising pH but remained stable at around 90%. The activation energy required for peptide degradation changes with pH, affecting the degradation pathway. Different pH values affect the actual degradation pathway used. Generally, each peptide has an optimal pH range. Within this pH range, the structure and the antioxidant activity are relatively stable. Therefore, FMP has better antioxidant activity under acid–alkaline conditions.

When food, medicine, and diet supplements are ingested orally, they pass through the gastrointestinal tract and are subject to the actions of digestive enzymes. During passage through the gastrointestinal tract, it is likely that their stabilities and bioactivities may be compromised. To assess how the antioxidant potential of FMP would be affected following exposure to gastrointestinal tract digestion, we exposed FMP to the enzymatic actions of pepsin and pancreatin to mimic the gastrointestinal tract environment [42,43]. In this study, DPPH radical scavenging activity increased, and the total antioxidant capacity of FRAP was basically unchanged. Further digestion with trypsin resulted in a decrease in antioxidant activity. Trypsin causes a decrease in surface hydrophobicity, disrupts a certain sequence length of the peptide, and decreases surface hydrophobicity, making it more hydrophilic and less able to react with lipid-soluble DPPH radicals [44]. Thus, the scavenging activity against the DPPH radical was decreased during the trypsin treatment. Indeed, we found that similar results were reported in duck meat, showing that peptides were hydrolyzed more completely after trypsin digestion, which led to an increase in hydrophilicity and made it more difficult for the peptides to react with lipid-soluble free radicals [45]. Previous studies found that the degradation of basic and aromatic amino acids may decrease the antioxidant activities of peptides [46]. Overall, the antioxidant activity of FMP decreased but remained high after simulated gastrointestinal digestion.

In recent years, evidence has emerged that oxidative stress plays a crucial role in the development and perpetuation of inflammation, and thus contributes to the pathophysiology of several debilitating diseases [47]. Inflammatory reactions and oxidative stress are involved in frequently observed enteric diseases of broiler chickens. The nutritional and economic consequences of mounting an inflammatory response in poultry are inversely related to body weight gain and overall performance [48]. Dietary antioxidants enhance growth performance and shield the body from reactive oxygen species (ROS) [49]. Canola Bioactive Peptides (CBPs) were added to broiler diets at 200 and 250 g/kg to enhance body weight gain (BWG) and reduce the feed-to-gain ratio (F/G) [50]. The effects of soybean bioactive peptide (SBP) on broilers F/G were noteworthy [51]. Body weight rise was seen in broiler diets supplemented with bioactive peptides derived from cottonseed [52]. All of the chicks in our investigation received sterile saline or polypeptide solution via gavage. The data showed that the HPG had significantly increased average daily weight gain compared to the DG, suggesting that peptides can prevent inflammation caused by OS. Although peptides are digested into smaller peptides or amino acids in the digestive system, amino acids generated by the hydrolysis of polypeptides may be responsible for this since amino acids form different polypeptide chains along with changes in amino acid types and branch chain locations. More significantly, research revealed that inflammation and growth performance were impacted by D and L aspartate [53]. In terms of amino acid balance, a polypeptide supplement can be a better option than a single amino acid.

The small intestine is the primary site for the absorption and transport of nutrients. Oxidative stress can directly harm the small intestine, which serves as the primary site for digestion and absorption [54]. The length of the villi is indicative of the small intestine’s capacity to assimilate nutrients. Only fully developed villus cells have the capability to assimilate nutrients. The intestinal crypts are specialized intestinal epithelial cells that possess secretion and absorption capabilities. They play a crucial role in replenishing the rapidly shedding epithelial cells of the small intestine’s villi. Over time, the crypts gradually become shallower. The V/C ratio provides a thorough assessment of the functional state of the small intestine. As the ratio increases, the mucosa undergoes repair, leading to improved digestion and absorption, as well as rapid growth. Conversely, a decrease in the ratio will have the opposite effect [55]. Prior research has demonstrated that antimicrobial peptides (ABPs), Fermented Soybean Meal (FSBM), and soybean bioactive peptide (SBP) have a substantial impact on the dimensions of villus height and crypt depth in the duodenum [51,55,56]. Consistent with previous research, this study found that the height of the duodenum in the DG was significantly lower compared to the CG. Additionally, the administration of polypeptide (25 mg/kg) was found to enhance the structure of the intestinal tissue in broilers, stimulate cell metabolism, and enhance the growth and maturity of villi cells. This may also be the reason why the DG recovered its growth performance.

Immune factors enhance the body’s resistance to disease by fostering the development of gut-dwelling beneficial bacteria and achieving flora balance. When the body is exposed to pathogens, the immune cells produce interferons to regulate the immune response to resist virus replication in the body. In this study, the rectal temperatures of chicks were measured four times after inducing OS for 24 h. The results show that within 12 h after HC and LPS injection, the anal temperature increased to varying degrees, and TNF-α, IL-1β, and IL-6 increased significantly, suggesting that there may be inflammatory reactions. However, compared with the DG, oral administration of FMP significantly reduced the anal temperature at 24 h in the MPG (15 mg/kg), and pro-inflammatory cytokines were significantly reduced. These results suggest that oral administration of exogenous FMP can reduce liver inflammation, significantly inhibit HC- and LPS-induced overproduction of inflammatory cytokines in the liver, and may help to ameliorate acute OS-induced swelling. Pro-inflammatory cytokines such as tumor necrosis factor (TNF)-α, interleukin (IL)-1β, and LPS are among the most potent NF-κB activators, acting on the extracellular receptors and initiating a relay of intracellular phosphorylation events, which co-ordinate signaling and conditional cell responses [57]. A report revealed that a polypeptide from the transcriptome of Turbinaria peltata significantly suppressed the production of LPS-challenged pro-inflammatory mediators and cytokines (including TNF-α, IL-6, and IL-1β), of which protective effects were associated with down-regulation of the NF-κB signaling pathway and alleviation of the expression of the inflammasome complex [58]. In addition, IL-10, an anti-inflammatory cytokine, is usually negatively correlated with the secretion of pro-inflammatory cytokines, such as IL-1β, TNF-α, and IL-6 [59,60]. Consistent with the previous study, the mechanism of the anti-inflammatory effect of FMP may be through the inhibition of the NF-κB signaling pathway and its mediated inflammatory response. Pro-inflammatory mediators in serum, pro-inflammatory cytokines (TNF-α, IL-6, and IL-1β), and anti-inflammatory cytokine (IL-10) in the liver were markedly altered with the administration of substances that induce OS but these changes were reversed by FMP.

The spleen, which plays a role in humoral immunity and can secrete certain immunological antibodies and produce lymphocytes, is the largest peripheral immune organ in chickens. The main function of the thymus is in cellular immunity. The bursal, which is primarily involved in humoral immunity and is the location of cell proliferation and differentiation that makes up the serum antibody system, is the distinct major immunological organ of fowl. To a certain extent, the state of development and proliferation of immune organs might indicate the overall level of immunity in the body [61]. Combined with serum antioxidant parameters at 5 d, we suppose that the chicks improved gradually and it was possible to accelerate the process for polypeptide administration. Therefore, the relative weight changes were not found. Moreover, the spleen, a central immune organ, has a certain blood storage function and there was still a significant difference between the DG and HPG in the spleen relative weight at 5 d. There was still a significant difference between the DG and HPG in the spleen relative weight at 5 d. Nevertheless, to the best of our knowledge, this is the first study to evaluate the mitigation effect of FMP on broilers exposed to HC and LPS; thus, no other studies could be compared. However, numerous studies have documented the beneficial effects of natural substances such as proanthocyanins and marine algal polysaccharides in reducing bursa of Fabricius injury in broilers. These natural products have been found to significantly enhance the relative weight of the bursa of Fabricius in broilers [62,63].

The antioxidant defense system eliminates free radicals produced during oxidative metabolism. An abundance of free radicals can harm large biological molecules like proteins and nucleic acids, leading to damage in tissues and cells. This disruption can affect the stability of the organism’s internal environment [64]. MDA, GSH, and SOD are commonly employed to assess the antioxidant state of tissues. MDA, a type of lipid peroxide that is generally stable, can be found in both blood and tissues. Its presence indicates the level of lipid peroxidation caused by oxygen free radicals, as well as the extent of oxidative damage in the organism indirectly. Oxidation and antioxidation remain in a state of dynamic stability in healthy individuals [61]. SOD and GSH-Px have a crucial function in protecting against oxidative stress. In this study, 15 mg/kg and 25 mg/kg of polypeptide could significantly increase T-SOD activity at 5 d but had little effect on GSH-Px activity, significantly decreasing MDA content in intestinal tissues and inhibiting lipid peroxidation.

## 5. Conclusions

The results show that FMP has good stability and supplementation with different amounts of FMP could alleviate oxidative stress damage to varying degrees. The HPG (25 mg/kg BW) had the best effect. These results suggest that FMP can alleviate the effects of oxidative stress on poultry. However, the mechanism is unknown. This study presents a theoretical foundation for the addition of maggot antioxidant peptide supplementation to protect the growth of broilers.

## Figures and Tables

**Table 1:** Changes in rectal temperature of chicks after oxidation stress among treatments (°C).

Groups	3 h	6 h	12 h	24 h
CG	40.89 ± 0.10 ^a^	40.67 ± 0.09 ^a^	40.98 ± 0.18 ^a^	40.90 ± 0.12 ^b^
DG	41.17 ± 0.13 ^a^	41.22 ± 0.12 ^ab^	41.54 ± 0.12 ^a^	40.93 ± 0.09 ^ab^
VG	41.21 ± 0.06 ^a^	41.21 ± 0.09 ^b^	41.34 ± 0.09 ^a^	40.73 ± 0.11 ^ab^
LPG	41.19 ± 0.16 ^a^	41.22 ± 0.11 ^b^	41.54 ± 0.11 ^a^	40.91 ± 0.09 ^ab^
MPG	41.33 ± 0.09 ^a^	41.18 ± 0.11 ^b^	41.08 ± 0.11 ^a^	40.54 ± 0.11 ^a^
HPG	41.25 ± 0.14 ^a^	41.23 ± 0.06 ^b^	41.28 ± 0.06 ^a^	40.95 ± 0.09 ^ab^
*p*-value	0.156	0.003	0.087	0.003

Notes: Means with similar superscripts letters in the same column indicate no significant differences (*p* > 0.05), and those with different superscripts letters in the same column indicate significant differences (*p* < 0.05). The values are presented as means ± SEM of 3 replications.

**Table 2:** Effect of FMP on growth performance of chicks.

Groups	Initial Weight (g)	Final Weight (g)	Average Daily Gain (ADG, g/d)
CG	59.68 ± 0.83 ^a^	149.36 ± 6.16 ^a^	11.21 ± 0.61 ^ab^
DG	59.38 ± 0.88 ^a^	127.77 ± 8.12 ^a^	8.23 ± 0.92 ^a^
VG	58.70 ± 0.52 ^a^	170.12 ± 5.32 ^a^	13.93 ± 0.71 ^b^
LPG	59.45 ± 0.65 ^a^	143.00 ± 10.50 ^a^	10.44 ± 1.89 ^ab^
MPG	57.44 ± 0.55 ^a^	146.02 ± 15.07 ^a^	10.87 ± 0.05 ^ab^
HPG	59.04 ± 0.93 ^a^	165.82 ± 19.11 ^a^	16.22 ± 3.52 ^b^
*p*-value	0.06	0.100	0.015

Notes: Means with similar superscripts letters in the same column indicate no significant differences (*p* > 0.05), and those with different superscripts letters in the same column indicate significant differences (*p* < 0.05). The values are presented as means ± SEM of 3 replications.

**Table 3:** Effect of FMP on the relative weight of immune organ of chicks (mg/g).

Groups	Bursa of Fabricius	Spleen
CG	4.29 ± 0.12 ^a^	1.22 ± 0.07 ^a^
DG	4.11 ± 0.16 ^a^	1.60 ± 0.06 ^b^
VG	4.33 ± 0.20 ^a^	1.17 ± 0.05 ^a^
LPG	3.72 ± 0.18 ^a^	1.54 ± 0.08 ^b^
MPG	3.72 ± 0.14 ^a^	1.35 ± 0.05 ^ab^
HPG	3.85 ± 0.15 ^a^	1.14 ± 0.04 ^a^
*p*-value	0.107	<0.001

Notes: Means with similar superscripts letters in the same column indicate no significant differences (*p* > 0.05), and those with different superscripts letters in the same column indicate significant differences (*p* < 0.05). The values are presented as means ± SEM of 3 replications.

**Table 4:** (**a**) Effect of FMP on serum indexes at 12 h. (**b**) Effect of FMP on serum indexes at 72 h. (**c**) Effect of FMP on serum indexes at 5 d.

(a)				
Groups	GSH-Px (μmol/L)	MDA (nmol/mL)	T-AOC (mM)	T-SOD (U/mL)
CG	233.78 ± 1.34 ^a^	3.90 ± 0.32 ^ab^	1.57 ± 0.06 ^b^	104.85 ± 0.87 ^a^
DG	238.54 ± 1.62 ^ab^	5.06 ± 0.31 ^b^	0.93 ± 0.11 ^a^	105.77 ± 4.27 ^a^
VG	238.61 ± 1.59 ^ab^	3.82 ± 0.12 ^a^	1.54 ± 0.07 ^b^	108.51 ± 1.17 ^a^
LPG	236.31 ± 1.55 ^ab^	4.75 ± 0.27 ^ab^	1.28 ± 0.06 ^b^	102.60 ± 2.47 ^a^
MPG	241.68 ± 3.16 ^b^	3.76 ± 0.32 ^a^	1.42 ± 0.04 ^b^	104.12 ± 1.16 ^a^
HPG	238.84 ± 1.37 ^ab^	4.17 ± 0.30 ^ab^	1.36 ± 0.04 ^b^	103.46 ± 1.74 ^a^
*p*-value	0.101	0.009	<0.001	0.462
**(b)**				
CG	302.49 ± 2.77 ^a^	4.00 ± 0.15 ^a^	0.77 ± 0.06 ^ab^	99.15 ± 0.72 ^a^
DG	315.53 ± 1.89 ^c^	6.72 ± 0.16 ^b^	0.74 ± 0.05 ^ab^	99.75 ± 1.61 ^a^
VG	308.31 ± 2.65 ^abc^	4.23 ± 0.15 ^a^	0.80 ± 0.03 ^ab^	102.21 ± 1.10 ^a^
LPG	314.17 ± 1.14 ^bc^	4.62 ± 0.38 ^a^	0.70 ± 0.17 ^a^	101.00 ± 0.87 ^a^
MPG	304.05 ± 4.36 ^ab^	4.03 ± 0.30 ^a^	0.83 ± 0.10 ^ab^	99.96 ± 1.69 ^a^
HPG	306.87 ± 2.61 ^abc^	4.38 ± 0.22 ^a^	0.93 ± 0.19 ^b^	98.14 ± 0.93 ^a^
*p*-value	0.003	<0.001	0.051	0.257
**(c)**				
CG	252.93 ± 1.77 ^a^	4.07 ± 0.32 ^b^	1.00 ± 0.05 ^ab^	98.91 ± 1.41 ^a^
DG	249.87 ± 1.64 ^a^	4.16 ± 0.09 ^b^	1.06 ± 0.09 ^ab^	97.43 ± 0.79 ^a^
VG	250.20 ± 3.40 ^a^	3.05 ± 0.08 ^a^	1.32 ± 0.07 ^b^	104.21 ± 1.7 ^ab^
LPG	250.20 ± 3.40 ^a^	3.34 ± 0.32 ^ab^	1.00 ± 0.07 ^a^	99.71 ± 2.03 ^a^
MPG	256.91 ± 2.81 ^a^	3.04 ± 0.08 ^a^	1.00 ± 0.06 ^ab^	108.59 ± 2.29 ^b^
HPG	250.94 ± 3.03 ^a^	3.59 ± 0.11 ^ab^	1.03 ± 0.06 ^ab^	109.91 ± 1.66 ^b^
*p*-value	0.35	<0.001	0.028	<0.001

Notes: Means with similar superscripts letters in the same column indicate no significant differences (*p* > 0.05), and those with different superscripts letters in the same column indicate significant differences (*p* < 0.05). The values are presented as means ± SEM of 3 replications.

**Table 5:** Effect of FMP on inflammatory cytokines of the liver (pg/mL).

Groups	IL-1β	IL-6	IL-10	TNF-α
CG	22.19 ± 0.35 ^c^	0.44 ± 0.01 ^b^	24.04 ± 4.87 ^ab^	2.37 ± 0.56 ^b^
DG	24.33 ± 0.21 ^d^	0.78 ± 0.01 ^c^	25.39 ± 4.23 ^d^	3.13 ± 0.07 ^b^
VG	19.97 ± 0.14 ^ab^	0.48 ± 0.02 ^b^	22.13 ± 2.56 ^bc^	0.54 ± 0.11 ^a^
LPG	18.92 ± 0.33 ^a^	0.25 ± 0.01 ^a^	22.63 ± 4.43 ^bc^	0.74 ± 0.07 ^a^
MPG	21.73 ± 0.64 ^c^	0.27 ± 0.02 ^a^	18.54 ± 1.55 ^a^	0.93 ± 0.05 ^a^
HPG	21.44 ± 0.33 ^bc^	0.42 ± 0.02 ^b^	20.53 ± 3.31 ^abc^	0.56 ± 0.08 ^a^
*p*-value	<0.001	<0.001	<0.001	<0.001

Notes: Means with similar superscripts letters in the same column indicate no significant differences (*p* > 0.05), and those with different superscripts letters in the same column indicate significant differences (*p* < 0.05). The values are presented as means ± SEM of 3 replications.

**Table 6:** (**a**) Effect of FMP on villus height, crypt depth, and VCR of duodenum. (**b**) Effect of FMP on villus height, crypt depth, and VCR of jejunum. (**c**) Effect of FMP on villus height, crypt depth, and VCR of the ileum.

(a)			
Groups	Villus Height (μM)	Crypt Depth (μM)	Villus Height to Crypt Depth Ratio (VCR)
CG	1319.52 ± 42.89 ^b^	97.99 ± 4.67 ^b^	14.36 ± 1.24 ^a^
DG	932.31 ± 40.63 ^a^	81.31 ± 3.23 ^ab^	11.42 ± 0.34 ^a^
VG	1183.96 ± 54.68 ^ab^	74.58 ± 3.34 ^a^	15.66 ± 1.83 ^a^
LPG	1088.3 ± 35.73 ^ab^	76.4 ± 4.14 ^a^	14.57 ± 2.14 ^a^
MPG	1150.11 ± 30.8 ^ab^	75.13 ± 3.16 ^a^	15.47 ± 0.95 ^a^
HPG	1275.57 ± 31.29 ^ab^	88.33 ± 6.91 ^ab^	15.05 ± 1.17 ^a^
*p*-value	<0.001	<0.001	0.602
**(b)**			
CG	909.38 ± 16.54 ^b^	77.20 ± 2.11 ^a^	11.04 ± 0.58 ^a^
DG	774.79 ± 27.75 ^b^	78.25 ± 2.21 ^a^	9.97 ± 0.83 ^a^
VG	779.70 ± 67.68 ^b^	65.78 ± 3.34 ^a^	9.88 ± 1.22 ^a^
LPG	850.55 ± 56.11 ^a^	71.04 ± 5.89 ^a^	11.47 ± 1.96 ^a^
MPG	802.83 ± 60.56 ^a^	78.12 ± 2.82 ^a^	11.71 ± 0.97 ^a^
HPG	952.38 ± 53.00 ^a^	72.91 ± 1.88 ^a^	13.1 ± 0.97 ^a^
*p*-value	<0.001	0.107	0.31
**(c)**			
CG	686.04 ± 18.40 ^ab^	70.20 ± 1.67 ^b^	10.47 ± 0.97 ^ab^
DG	622.69 ± 15.46 ^bc^	68.05 ± 2.30 ^ab^	9.00 ± 0.39 ^ab^
VG	530.51 ± 28.12 ^d^	68.12 ± 1.56 ^ab^	8.32 ± 0.67 ^a^
LPG	591.67 ± 16.71 ^cd^	63.19 ± 1.96 ^ab^	9.61 ± 0.44 ^ab^
MPG	607.55 ± 9.85 ^bcd^	62.22 ± 2.25 ^a^	9.35 ± 0.64 ^ab^
HPG	734.47 ± 9.74 ^a^	64.85 ± 1.38 ^ab^	11.44 ± 0.35 ^b^
*p*-value	<0.001	0.015	0.024

Notes: Means with similar superscripts letters in the same column indicate no significant differences (*p* > 0.05), and those with different superscripts letters in the same column indicate significant differences (*p* < 0.05). The values are presented as means ± SEM of 3 replications.

## Data Availability

Data are contained within the article.

## Supplementary Materials

The following supporting information can be downloaded at: https://www.mdpi.com/article/10.3390/vetsci11100470/s1, Supplementary Table S1: Abbreviations.
