# Supplementary material for: Stability of Fly Maggot Peptides and Its Alleviating Effect on Lipopolysaccharide Combined with Hemocoagulase Oxidative Stress in Arbor Acres Chicks
Source: Vet Sci. 2024 Oct 1;11(10):470. doi: 10.3390/vetsci11100470 (PMC11511490; doi:10.3390/vetsci11100470)
Supplement: Supplementary file 1 [file vetsci-11-00470-s001.zip › vetsci-3210358-supplementary.pdf]

Supplementary Table S1. Abbreviations

Abbreviated name	Full name
FMP	Fly maggot peptide
LPS	Lipopolysaccharide
HC	Hemocoagulase
FRAP	Ferric ion-reducing antioxidant power
DPPH	2,2-Diphenyl-1-picrylhydrazyl
TNF-α	Tumor necrosis factor-α
IL-1β	Interleukin-1β
IL-6	Interleukin-6
IL-10	Interleukin-10
MDA	Malondialdehyde
T-AOC	Total antioxidant capacity
T-SOD	Total superoxide dismutase
GSH-PX	Glutathione peroxidase
CG	Control group
DG	Damage group
VG	Vitamin C group
LPG	5 mg/kg polypeptides group
MPG	15 mg/kg polypeptides group
HPG	25 mg/kg polypeptides group
VH	Villus height
CD	Crypt depth
VCR	Villus height to crypt depth ratio
